# Biosynthesis of the *Escherichia coli* K1 group 2 polysialic acid capsule occurs within a protected cytoplasmic compartment

**DOI:** 10.1111/j.1365-2958.2008.06231.x

**Published:** 2008-06

**Authors:** Susan M Steenbergen, Eric R Vimr

**Affiliations:** Laboratory of Sialobiology and Comparative Metabolomics, Department of Pathobiology, University of Illinois at Urbana-Champaign Urbana, IL 61802, USA

## Abstract

Capsular polysaccharides are important virulence determinants in a wide range of invasive infectious diseases. Although capsule synthesis has been extensively investigated, understanding polysaccharide export from the cytoplasm to the external environment has been more difficult. Here we present the results of a novel protection assay indicating that synthesis and export of the *Escherichia coli* K1 group 2 capsular polysialic acid (K1 antigen) occur within a protected subcellular compartment designated the sialisome. In addition to the polymerase encoded by *neuS*, localization and complementation analyses indicated that the sialisome includes the accessory membrane protein NeuE. The requirement for NeuE was suppressed by overproducing NeuS, suggesting that NeuE functions by stabilizing the polymerase or facilitating its assembly in the sialisome. Although an interaction between NeuE and NeuS could not be demonstrated with a bacterial two-hybrid system that reconstitutes an intracellular cell-signalling pathway, interactions between NeuS and KpsC as well as other sialisome components were detected. The combined results provide direct evidence for specific protein–protein interactions in the synthesis and export of group 2 capsular polysaccharides under *in vivo* conditions. The approaches developed here will facilitate further dissection of the sialisome, suggesting similar methodology for understanding the biosynthesis of other group 2 capsules.

## Introduction

Microbial exopolysaccharides, defined here as cell-adherent capsules or excreted, unattached or loosely attached slime, are used as additives in a wide range of food and cosmetic products, medical replacements in ocular and joint surgeries, and are essential components of microbial biofilms (Sutherland, 1998; 2001). Some exopolysaccharides are also important virulence determinants in bacterial or fungal invasive disease where, as capsules surrounding the microbial cell surfaces, they function as ligands for cell adhesion or modulators in the avoidance or inhibition of innate and acquired immunity (Vimr *et al.*, 2004). Capsular polysaccharides conjugated to protein carriers conferring T-cell dependency are among the most effective anti-capsule vaccines in current medical use: *Streptococcus* spp., group b *Haemophilus influenzae*, and all groups except group B *Neisseria meningitidis*, with more anti-capsule vaccines in development (Robbins and Schneerson, 2004). Supporting these applied uses of exopolysaccharides are basic studies to understand the molecular details of capsule biosynthesis, which we define here as the processes of intracellular polysaccharide synthesis and export (secretion or translocation) to the external environment. In many cases, the linkage of polysaccharides to the outer membrane of Gram-negative bacteria or cytoplasmic membrane of Gram-positive organisms is unknown (Whitfield, 2006).

*Escherichia coli* is a model organism for investigating bacterial exopolysaccharide function and biosynthesis. Different types of *E. coli* capsules have been subdivided into one of four biosynthetic groups (Whitfield, 2006). After synthesis of the required nucleotide sugar(s) unique to a given polymeric structure, functionally diverse glycosyltransferases catalyse synthesis of homo- or heteropolysaccharides that are exported to the cell surface by ATP-binding cassette-like (ABC) transporters or Traffic ATPases, in the case of groups 2 and 3 capsules, or by Wzy-dependent machineries required for groups 1 and 4 export. In the case of groups 2 and (probably) 3 polysaccharides, some sugar chains are anchored to the cell membrane by esterification to phospholipid, further distinguishing them from the lipid A-linked somatic or O antigens of lipopolysaccharide. Mechanistic distinctions are also based on the genetic regulation and map positions of the capsule gene clusters. We suggest a fifth group of polysaccharides in which the glycosyltransferase (polymerase) simultaneously functions as the translocase. This system, where the polymerase is also the cytoplasmic membrane exporter, has been inferred for the biosynthesis of Gram-positive and -negative polyglucosamines and hyaluronans (Wang *et al.*, 2004; Fluckiger *et al.*, 2005; Tlapak-Simmons *et al.*, 2005). Despite our understanding of groups 1 and 4 Wzy-dependent export systems, transport of group 2 capsular polysaccharides has been a more difficult process to study (Whitfield, 2006). This difficulty stems in part from problems in reconstituting the translocation process after subcellular fractionation (Vimr and Steenbergen, 1993).

Attempts to reconstitute polysialic acid (PSA) synthesis and export using inverted (inside-out) membrane vesicles and an *in vitro* protection assay indicated that only a relatively small and temporally constant fraction of the total PSA synthesized by this system was resistant to endo-N digestion, implying a failure of the system to export polysialic acid (Vimr and Steenbergen, 1993). Endo-N is a K1-specific bacteriophage-derived depolymerase that cleaves α2,8-linked sialic acid residues of PSA chains ≥ 7 monomers in length (Petter and Vimr, 1993). Our *in vitro* results suggested that some factor or physical property of the system was lost during subcellular fractionation which prevented efficient export of PSA despite continual polymer synthesis in the presence of exogenously added sugar nucleotide donor cytidine 5′-monophospho *N*-acetylneuraminic acid (CMP-Neu5Ac) and ATP, and thus continual sensitivity to endo-N, a homotrimer composed of 76 kDa subunits. Although the ‘flippase’ activity of Wzx found in groups 1 and 4 systems has been demonstrated *in vitro* (Rick *et al.*, 2003), we are unaware of any successful reconstitution of group 2 polysaccharide export. We concluded from our *in vitro* protection studies that PSA biosynthesis involves essential protein–protein, protein–polysaccharide or protein–lipid interactions that are disrupted during fractionation. In the current study, we describe an *in vivo* investigation of PSA biosynthesis that provides the first direct evidence for a novel subcellular compartment, which we propose to designate the sialisome. Similarly privileged cytoplasmic compartments may exist in other group 2 polysaccharide biosynthetic systems.

## Results and discussion

### Mechanisms and models of capsular polysaccharide biosynthesis

**Fig. 1:**
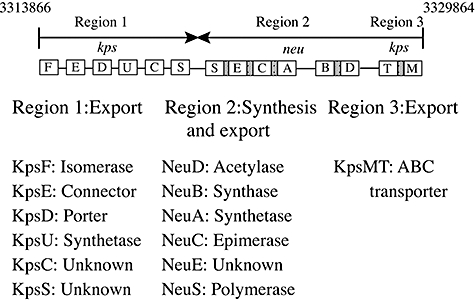
Genetic organization of the group 2 capsular polysialic acid gene cluster in *E. coli* K1. Biosynthetic genes (not drawn to scale) are shown as open boxes with transcriptional directions indicated by the horizontal arrows above. The chromosomal positions in bp of the start of region 1 and the beginning of region 3 are given by the numbers at left and right, respectively, as taken from the *E. coli* K1 strain RS218 genomic DNA sequence (http://www.genome.wisc.edu/sequencing/rs218.htm). Dotted rectangles indicate translationally coupled open reading frames. The known or presumed gene functions in capsule biosynthesis are given below the figure, as summarized in the text and by Steenbergen and Vimr (2003).

**Fig. 2:**
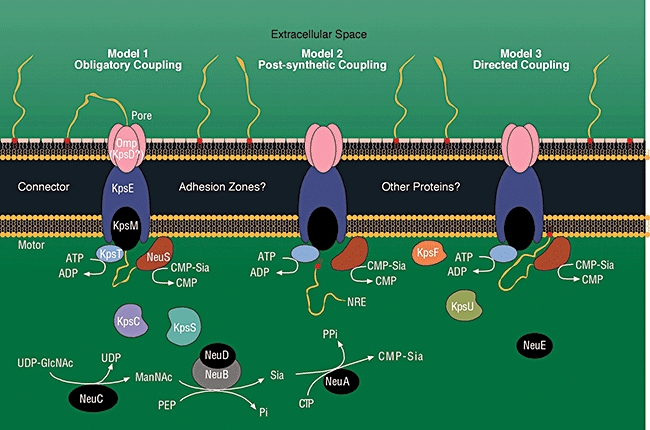
Three models of group 2 polysaccharide biosynthesis. Known or suspected functions of *kps* and *neu* gene products are defined in the legend to Fig. 1 and text. Gold ribbons represent PSA with red squares indicating phospholipids esterified at the reducing ends. Sialic acid monomers are polymerized by NeuS-catalysed additions to the non-reducing end (NRE). Phospholipids are shown in gold comprising both leaflets of the inner (cytoplasmic) membrane, as well as the inner leaflet of the outer membrane. Lipopolysaccharide molecules in the outer leaflet of the outer membrane are shown as white rectangles. The periplasmic space (with the peptidoglycan layer omitted for convenience) is indicated by the dark-blue colour, with KpsE postulated as the connector from the KpsMT ABC exporter to the Omp or KpsD outer membrane pore. Note that both KpsM and KpsT function as homodimers, omitted for convenience from the diagram. In addition to the *kps* and *neu* gene products indicated, other proteins may be involved in polysaccharide translocation or assembly of the export apparatus. Details of the various models are described in the text. GlcNAc, *N*-acetylglucosamine; ManNAc, *N*-acetylmannosamine; PEP, phosphoenolpyruvate; Sia, *N*-acetylneuraminic acid (sialic acid); PPi, pyrophosphate; Omp, outer membrane protein.

Groups 2 and 3 capsule biosynthetic gene clusters include loci unique to the structural requirements of a given polysaccharide (region 2) and sets of shared regions 1 and 3 genes encoding the ABC transporters and several proteins with unclear functions which are nonetheless required for polymer export (Fig. 1). We suggest three possible biosynthetic mechanisms to explain the synthesis and export of group 2 capsules. In model 1 (Fig. 2), synthesis and export are obligatorily coupled processes. In such a coupled system, loss of export function would result in the concomitant loss of capsule synthesis, as observed for the *E. coli* K30 polysaccharide that fails to accumulate in a mutant lacking the outer membrane protein Wza (Whitfield and Paiment, 2003). By contrast, group 2 export-defective mutants accumulate full-length intracellular polysaccharides in all regions 1 and 3 or *neuE* mutants so far investigated (reviewed in Vimr *et al.*, 2004).

Model 2 indicates a two-step process in which synthesis occurs independently of export, and where phospholipid modification of the reducing terminus may trigger activation of or signal PSA entry into the export apparatus. This hypothesis is consistent with the accumulation of full-length polysaccharides in group 2 mutants with region 1 or 3 export defects, potentially explaining how a conserved export apparatus transports structurally distinct polysaccharides by recognizing a common chemical modification or secondary structural feature (reviewed in Vimr *et al.*, 2004). Because the products of the region 1 *kpsC* and *kpsS* genes are homologous with certain enzymes of lipid metabolism, and because mutations in these genes were reported to cause accumulation of intracellular group 2 polysaccharides lacking the reducing terminal phospholipid modification (Bronner *et al.*, 1993; Frosch and Muller, 1993), model 2 seemed to be supported. However, the experimental observations supporting model 2 are inconsistent with the phenotypes of *E. coli* K1 *kpsC* or *kpsS* mutants, which accumulate lipidated PSA, suggesting that lipidation is insufficient for group 2 capsule export (Cieslewicz and Vimr, 1996). The explanation for the experimental discrepancies is unclear, but suggests that neither KpsS nor KpsC is required for lipid addition, calling model 2 into question. This conclusion is supported by a recent report that group B meningococcal *kpsS* (*lipB* or *ctrC*) and *kpsC* (*lipA* or *ctrD*) mutants also accumulate lipidated, intracellular PSA (Tzeng *et al.*, 2005). The reason why previous studies did not detect polysaccharide lipidation in *kpsC* or *kpsS* mutants in the *E. coli* K5 or meningococcal systems may have been failure to control for the heterologous or non-stoichiometric expression systems used in these studies, or relatively low sensitivity of the detection methods. In contrast, studies detecting lipidation of PSA in *E. coli* K1 and group B meningococcal *kpsS* and *kpsC* mutants were carried out with strains containing single-copy capsule gene clusters and enzymatic or structural methods that unambiguously detected phospholipid attachment.

Model 3 is a hybrid of models 1 and 2 and predicts that while polysaccharide synthesis occurs in the absence of transport, only those polymers synthesized in the presence of an intact biosynthetic complex are exported. In the extreme case, assembly of the functional translocase would occur only when triggered by nascent group 2 polysaccharide. Model 3 suggests intimate contact between synthetic and export components of group 2 biosynthetic complexes, consistent with the reduced polymerase activities observed for all regions 1, 3 and *neuE* mutants (reviewed in Vimr *et al.*, 2004). This phenotype indicates that polymerase catalytic efficiency is affected by the operation or structure of the export apparatus, which is qualitatively similar to the feedback mechanism inferred for group 1 exopolysaccharides.

### Polysialic acid synthesis and export occur within a protected subcellular compartment operationally defined as the sialisome

**Fig. 3:**
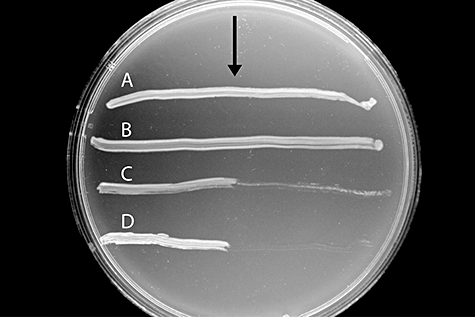
PSA biosynthesis is protected from endo-N *in vivo*. Approximately 75 μl of K1-specific phage K1F (1 × 10^10^ pfu ml^−1^ phage stock solution) was streaked (arrow) down the middle of the plate with a sterile wooden stick, and bacteria were dragged across the phage from left to right. The plate was photographed after overnight incubation at 37°C. A, *E. coli* K-12 strain MC4100; B, acapsular mutant EV93; C, EV36 harbouring pEndo-N; D, EV36.

To distinguish between models 2 and 3 (Fig. 2), we transformed the wild-type K1 strain EV36 with plasmid expressing endo-N under control of a *lac* promoter and assayed for PSA expression by sensitivity to phage K1F. Endo-N is a PSA-specific depolymerase that is also the tailspike protein for K1-specific bacteriophage, where it functions as a necessary phage organelle for infection (Vimr *et al.*, 1984; Petter and Vimr, 1993). As shown in Fig. 3, the transformed strain was as susceptible to phage infection as the EV36 (wild type) control. As productive lytic infection requires an extracellular PSA receptor, the results suggested that polymer synthesis and all of the steps necessary for or leading to translocation occur within an endo-N-inaccessible compartment, operationally defined here as the sialisome, and providing direct experimental support for model 3 (Fig. 2). Similar results were obtained whether IPTG was present in the plates or not, or whether the recipient was co-transformed with pRep4, which carries the *lacI* repressor, indicating that endo-N gene expression was not limiting.

**Fig. 4:**
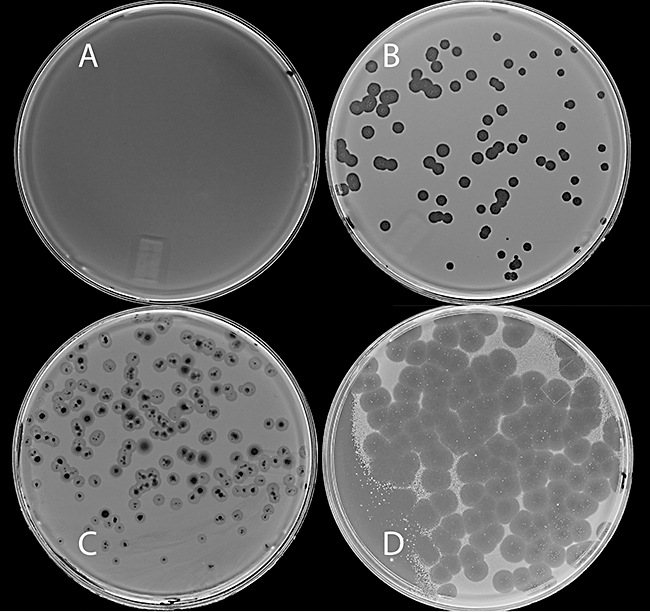
Effects of recombinant endo-N overproduction on K1-specific phage sensitivity. Bacteria were grown to early stationary phase with appropriate drugs and IPTG added where indicated to a final concentration of 1 mM to induce endo-N production when cells reached an A_600_ of 0.3. Bacteria (0.15 ml) were plated with approximately 85 K1F plaque forming units in top agar on LB and incubated at 37°C overnight prior to photography. A. Acapsular mutant EV93. B. EV36 wild type. C. EV36 harbouring pRep4 and pEndo-N, uninduced. D. EV36 harbouring pRep4 and pEndo-N, induced and plated to LB containing 1 mM IPTG.

As shown in Fig. 4B, EV36 had the same efficiency of K1F plating as its uninduced (Fig. 4C) or induced (Fig. 4D) derivatives harbouring pRep4 and pEndo-N. As expected, acapsular mutant EV93 (*kpsC*) did not support phage infection (Fig. 4A) because it lacks surface capsular polysaccharide, although it accumulates intracellular, unexported PSA in centrally located compartments termed lacunae to indicate their electron transparency (Cieslewicz and Vimr, 1996). Although plaque size differed under the three infection conditions (Fig. 4B–D), the results indicated that capsule was produced independently of the amount of intracellular endo-N, from none (Fig. 4B) to approximately 2% of the intracellular protein concentration of induced cells harbouring pRep4 and pEndo-N (Fig. 4D). We suggest that recombinant endo-N released by phage lysis accounts for the morphological plaque differences by altering cell-surface capsule or PSA released by disrupted cells. However, note that plaque size in the induced strain (Fig. 4D) was at least twice that of wild type (Fig. 4B) despite a reduced host growth rate caused by enzyme overproduction, indicating that even a massive accumulation of intracellular endo-N did not inhibit capsule biosynthesis. This is in contrast to *in vitro* results in which endo-N was shown to effectively degrade nascent PSA (Steenbergen *et al.*, 1992; Vimr and Steenbergen, 1993).

**Fig. 5:**
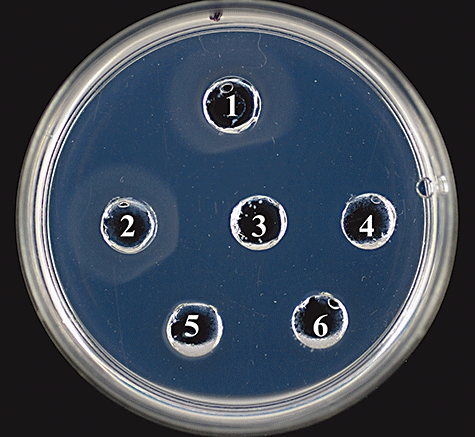
Production of endo-N in uninduced and induced EV36 harbouring pRep4 and pEndo-N. Bacteria (20 ml) were grown to early stationary phase with appropriate drugs and pelleted by centrifugation. The pellets were washed in phosphate-buffered saline (PBS), re-suspended in PBS to final volume of 0.5 ml and disrupted by sonication. The sonicate was centrifuged for 15 min at 35 000 *g* and 25 μl amounts of supernatants either loaded with an equal volume of PBS into wells of a radial immunodiffusion plate containing 10% (v/v) anti-polysialic acid antibody, or pre-incubated for 15 min at 37°C with an equal volume of induced or uninduced extract from EV36 harbouring pRep4 and pEndo-N prior to loading. Wells 1–4 contained extracts from EV36, EV93, EV36 pRep4 pEndo-N and induced EV36 pRep4 pEndo-N respectively. Wells 5 and 6 contained EV93 extracts pre-incubated with uninduced or induced extracts of EV36 harbouring pRep4 and pEndo-N respectively. Precipitin halos indicate diffusion of PSA and reaction with the antibody. The photograph was taken after overnight incubation at 32°C and 1 day at 4°C.

To demonstrate that both basal and induced endo-N productions by EV36 transformed with pRep4 and pEndo-N were sufficient for PSA degradation, we carried out radial immunodiffusion against anti-PSA antibody recognizing PSAs ≥ 8–10 sialic acid residues. If endo-N is present, PSA is degraded to oligomers < 8 residues in length that do not react with antibody. As shown in Fig. 5, extracts of both wild type (well 1) and mutant strain EV93 (well 2) contained PSA, as evident by the precipitin halos surrounding the respective wells. Note that strain EV93 PSA is derived from unexported, intracellular PSA whereas the wild-type antigen comes from capsular polysaccharide released during cell disruption (Cieslewicz and Vimr, 1996). In contrast, PSA from EV36 was degraded in the presence of basal or induced amounts of endo-N, resulting in the absence of precipitin halos (Fig. 5, wells 3 and 4 respectively). To directly demonstrate the susceptibility of intracellular PSA to endo-N, extracts of EV93 were mixed 1:1 with those of uninduced (Fig. 5, well 5) or induced (Fig. 5, well 6) extracts from EV36 harbouring pRep4 and pEndo-N. The absence of PSA antigen under either condition indicates that the expression of even a basal amount of endo-N is sufficient to depolymerize intracellular PSA.

**Fig. 6:**
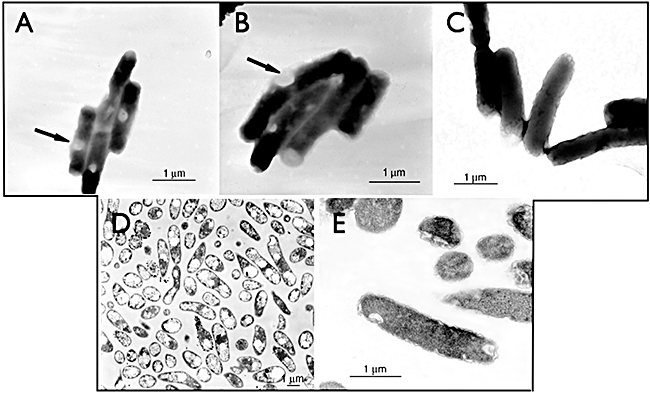
Susceptibility of intracellular PSA to endo-N digestion. A and B. TEM of PSA lacunae (arrows) in *E. coli* K1 mutant EV93 whole mounts. C. TEM of strain EV93 transformed with pEndo-N. D. TEM of thin-sectioned strain EV93. E. TEM of thin-sectioned strain EV93 transformed with pEndo-N.

An alternative argument for the results shown in Figs 3 and 4 is that endo-N is inactive when expressed intracellularly. To show that endo-N is active *in vivo*, we investigated PSA stability by transmission electron microscopy (TEM) of intact and thin-sectioned cells. We had previously shown by TEM that *E. coli* K1 export mutants accumulate lipidated PSA in intracellular lacunae unbounded by a membrane and displaying morphologies unique to a particular export defect (Cieslewicz and Vimr, 1996; 1997), indicating that *E. coli* normally lacks depolymerase(s) for turnover of the PSA that accumulates in these export mutants. In the case of strain EV93, the unexported PSA tends to accumulate in centrally located lacunae evident in thin sections (Cieslewicz and Vimr, 1996), a result that was confirmed independently for this study (Fig. 6D). The PSA in these accretion bodies bear terminal phospholipids that we assume result in the intracellular micelles formed by otherwise highly soluble and electrostatically charged polysaccharides. Lacunae thus represent intracellular organelles resulting from synthesis of full-length, lipidated PSA that either does not enter the translocation pathway or is aborted during biosynthesis. When whole mounts of negatively stained EV93 were examined by TEM, lacunae were apparent as bubble-like organelles in the intact cells (Fig. 6A and B). In contrast, lacunae were absent in EV93 transformed with pEndo-N with or without IPTG induction (Fig. 6C), indicating that basal endo-N expression was sufficient to degrade intracellular PSA. Similarly, lacunae were not detected when EV93 harbouring pEndo-N was examined by TEM of thin-sectioned samples (Fig. 6E). Taken together, the results of the intracellular endo-N protection assay demonstrate *in vivo* depolymerase activity against intracellular PSA, supporting the conclusion that wild-type PSA synthesis and export occur within an environment inaccessible to endo-N. Note that this operational definition of the sialisome does not preclude the concept of a kinetic compartment, in which the synthesis of PSA is so rapid that endo-N, even at induced concentrations, does not have time to degrade nascent PSA. However, because even one intra-chain clip would be expected to disrupt biosynthesis, we think it is more likely that the sialisome represents the functional biosynthetic complex that has been previously shown to involve multiple protein–protein interactions (McNulty *et al.*, 2006). More important, our results tend to exclude model 2 by favouring the directed coupling of PSA biosynthesis to its export shown in model 3 (Fig. 2).

### Complementation and suppression of the *neuE* translocation defect by *neuS* overexpression

**Fig. 7:**
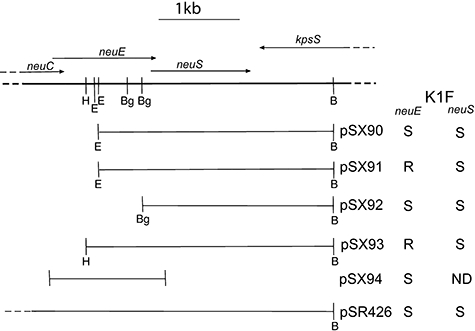
Complementation of *neuE* and *neuS* mutants. Strains EV725 (*neuE*) and EV136 (*neuS*) were transformed with the indicated plasmids and sensitivity to phage K1F was assayed by cross-streak and plaque assays. S (sensitive) and R (resistant) indicate relative susceptibility to phage infection. H, E and Bg indicate HindIII, EcoRI and BglII restriction endonuclease sites respectively. Note that the fragment in pSX90 is expressed in the same orientation as the vector *lacZ* promoter, whereas it is the opposite orientation in pSX91.

**Fig. 8:**
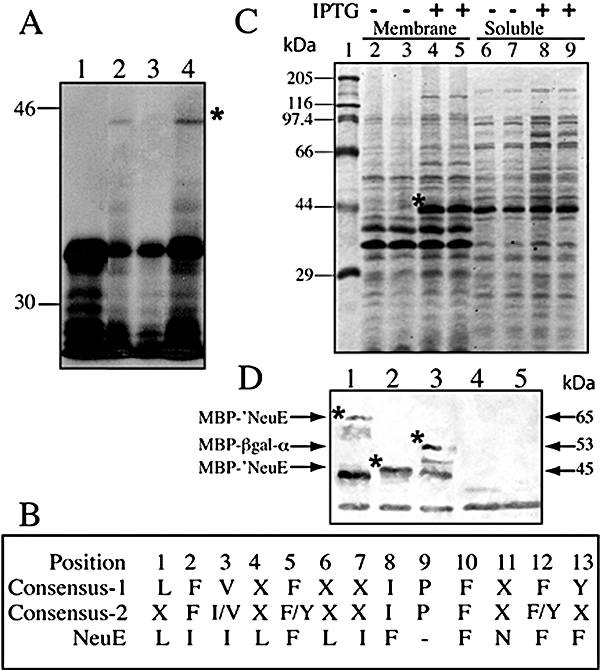
Overproduction of NeuS and membrane anchor function of the NeuE PIRS domain. A. Maxi-cell analysis of *neuS* expression in strain HB101 harbouring the indicated plasmids. Lane 1, pUC18; lane 2, pSX90; lane 3, pSX91; lane 4, pSX92. Asterisk indicates the overproduced NeuS polypeptide. B. The consensus-1 and-2 PIRS domains are taken from Zimmerman and Robbins (1993) and Datta and Lehrman (1993) respectively. One-letter amino acid designations are shown, where X can be any amino acid. Note that NeuE lacks the conserved proline residue at position 9 (Steenbergen *et al.*, 1992). C. Two independent EV725 (*neuE::kan*) subclones transformed with pSX101 expressing MBP fused to the PIRS domain (even or odd lanes respectively) were disrupted by sonication and fractionated into total membrane and soluble fractions by differential centrifugation. Overproduction of the 45 kDa fusion polypeptide is evident in the membrane fractions from induced transformants (asterisk). Numbers at left correspond to the molecular masses (kDa) of polypeptide markers shown in lane 1. D. Western blot analysis of total cell extracts from strain EV725 harbouring pSX104 (lane 1), pSX101 (lane 2) or pMal-c2 (lane 3) induced with IPTG prior to fractionation, electrophoresis and detection of MBP epitopes with anti-MBP antibody. Lanes 4 and 5 show results of the analysis with extracts from uninduced cells harbouring pSX101 or pMal-c2 respectively. The relevant bands (asterisks), designated at left, are defined in the text, with their respective molecular masses given at the right of the figure.

We previously showed that inactivation of *neuE* results in reduced NeuS activity and accumulation of intracellular PSA, a translocation-defective phenotype similar in all regions 1 and 3 mutants so far investigated, suggesting that NeuE is not obligatory for PSA synthesis but is required for export (Vimr and Steenbergen, 1993; Vimr *et al.*, 2004). However, a recent study indicated that NeuE was part of a NeuS complex, suggesting that NeuE might function directly in synthesis (Andreishcheva and Vann, 2006). Because our previous studies could not distinguish between a polar effect of the *neuE* mutation in EV725 on *neuS* expression and a direct effect of the mutation on translocation, we carried out a series of complementation experiments to distinguish between these possibilities. As shown in Fig. 7, complementation *in trans* with *neuE*^+^ carried by pSX94 restored the unencapsulated recipient to an encapsulated phenotype, as detected by sensitivity of the transformant to K1-specific lytic phage infection. Surprisingly, plasmid pSX92, which carries a truncated copy of *neuE* but has *neuS*^+^ oriented in the same direction as the *lacZ* promoter, complemented EV725 (Fig. 7), suggesting that *neuS* overexpression suppresses the requirement for *neuE*. This conclusion was supported by maxi-cell analysis and the complementation pattern for plasmids pSX90 and pSX91 with oppositely oriented *neuS* (Figs 7 and 8A). As expected, plasmid pSR426, which carries all of regions 2 and 3, complemented EV725 (Fig. 7). In agreement with the translational start of *neuE* overlapping the upstream *neuC* gene (Andreishcheva and Vann, 2006), plasmid pSX93 did not complement strain EV725 (Fig. 7).

NeuE has been shown to include a putative C-terminal polyisoprenoid recognition signal (PIRS) that could play a role in polyprenol binding during PSA synthesis or translocation (Fig. 8B) (Steenbergen *et al.*, 1992; Zhou & Troy, 2003, 2005). However, NeuS activity is not eliminated in a *neuE* null mutant, indicating that PIRS is not obligatory for polymerization (Vimr *et al.*, 2004), a conclusion supported by the analyses shown in Figs 7 and 8A. To determine if the NeuE PIRS domain functions as a membrane anchor, and thus a possible structural role consistent with the export defect of a *neuE* mutant (Vimr *et al.*, 2004), we carried out localization experiments with maltose binding protein (MBP) fused to the domain.

Depending on the programme used, *neuE* is predicted to encode a polypeptide with one to five membrane-spanning regions (Table 1). Each of the nine programmes identified the putative C-terminal PIRS domain while four of them uniquely identified PIRS as the only membrane-spanning region (Table 1). To confirm that PIRS functions as membrane anchor, we fused the BglII-BamHI fragment from pSX92 to the MBP polylinker in plasmid pMal-c2, generating plasmid pSX101 expressing an N-terminal MBP lacking its leader peptide sequence fused to the PIRS domain and the last 25 amino acid residues of NeuE. As shown in Fig. 8C (lanes 4 and 5), an overproduced polypeptide of the expected molecular weight was localized to the membrane fraction after differential centrifugation and denaturing polyacrylamide gel electrophoresis, indicating that PIRS anchored a soluble polypeptide to what we presume is the inner membrane. No overproduced polypeptide was detected in the soluble fraction (Fig. 8C, lanes 6–9), indicating efficient localization of the putative fusion to the membrane. However, the PIRS anchor is unlikely to span the membrane because nine of the 25 amino acid residues following it to the C-terminus are positively charged and therefore unlikely to cross into the periplasm. On the basis of secondary structural predictions (MacVector 9.0.2), we further suggest that the PIRS domain is unlikely to be an alpha helix, and more likely to be an amphipathic beta sheet reminiscent of the proposal for a similar anchoring domain in hyaluronan synthase (Heldermon *et al.*, 2001). Thus, while the exact function of NeuE is still unclear, our results indicate that it is not obligatory for PSA synthesis, although necessary for export. We have not yet tested whether deletion of this domain affects the export function.

**Table 1:** Computer-assisted analysis of NeuE TM regions.

	No. of NeuE	NeuE orientation	
Program	TM regions	N-terminus	C-terminus
das	3	ND	ND
hmmtop 2.0	4	i	i
pred-tmr 2	1	ND	ND
split 3.5	1	ND	ND
tmap	3	ND	ND
TM-Finder	3	ND	ND
tmhmm 2.0	1	o	i
TMpred	5	i	o
TopPred II	1	i	o

ND, not determined; o, outside (periplasmic); i, inside (cytoplasmic).

Controls with anti-MBP confirmed that the overproduced fusion polypeptide included the expected MBP epitope(s). Thus, the pMal-c2 vector alone synthesized the expected 53 kDa polypeptide (MBP-β-gal-α), whereas fusion of an almost full-length copy of *neuE* (pSX104) synthesized the expected 65 kDa polypeptide resulting from truncation of the *lacZ*α peptide of the vector by cloning the EcoRI-HindIII fragment from pSX90 into the pMal-c2 polylinker (Fig. 8D, lanes 3 and 1 respectively). The polyclonal anti-MBP antibody also detects lower-molecular-weight bands probably arising from proteolysis, including prominent bands near the 43 kDa size of native MBP, and a band common to strains independent of IPTG induction (Fig. 8D, lanes 1–5), representing an *E. coli* antigen recognized by the polyclonal antibody preparation. Induction of the strain expressing the *malE*-BglII-BamHI fragment from pSX90 (pSX101), which codes for the PIRS domain and last 25 amino acid residues of NeuE, stably produced the expected 45 kDa fusion polypeptide (Fig. 8D, lane 2) that was observed in the membrane fraction of induced cells (Fig. 8C, lanes 4 and 5). The results of this Western blot analysis confirm the identity of the overproduced membrane polypeptide as MBP fused to the NeuE PIRS domain. We conclude that this domain functions as NeuE membrane anchor, as predicted by the results in Table 1.

### Predictions of the sialisome concept

Involvement of the sialisome in PSA synthesis and translocation predicts interactions between NeuS and the export apparatus. Silver and colleagues demonstrated that KpsM and KpsT function as the inner membrane exporter and ATPase components of the group 2 ABC transporter respectively (Pigeon and Silver, 1994; Bliss *et al.*, 1996). KpsE is thought to include a large periplasmic domain that might connect the ABC transporter to the outer membrane, possibly involving KpsD as chaperone or outer membrane pore (Arrecubieta *et al.*, 2001; McNulty *et al.*, 2006). KpsC and KpsS share homology with enzymes of lipid metabolism and appear from Western blot analyses to interact with the inner membrane (Rigg *et al.*, 1998). The requirement or function in PSA biosynthesis of KpsF (isomerase) and KpsU (CMP-ketodeoxyoctonate, KDO, synthetase) is uncertain, although in the K5 system KDO is thought to link the capsular polysaccharide chain to phospholipid (Whitfield, 2006 and references cited therein). Except for the ABC transporter, the exact functions of other *kps* gene products are unclear, although mutation of most region 1 or region 3 and *neuE* genes results in an acapsular phenotype and diminution of *in vitro* NeuS activity (reviewed in Vimr *et al.*, 2004).

To develop an *in vivo* approach for investigating PSA synthesis and export, we used a two-hybrid bacterial system for the detection of both homo- and heterotypic protein–protein interactions. Daines and Silver (2000) previously used a Lex-based system to identify an interaction between NeuD and NeuB, implicating NeuD as a stabilizer or activator of the synthase (Fig. 2), a function recently supported by protein engineering of the homologous group B streptococcal NeuD (Lewis *et al.*, 2006). This conclusion is consistent with the function of NeuD as a monomeric sialic acid transacetylase (Steenbergen *et al.*, 2006), in which case direct interaction with NeuB might ensure efficient acetylation during the synthase-catalysed condensation of ManNAc with PEP to produce sialic acid. As an alternative to the Lex system, which requires the use of special strains to prevent possibly conflicting homodimeric interactions, we tested the applicability of the *Bordetella pertussis* adenylate cyclase system (Karimova *et al.*, 1998; 2000). This system depends on reconstitution of an active adenylate cyclase and subsequent transcriptional amplification of cAMP-dependent promoters, such as *lac* or *mal* expressed in an *E. coli cya* host. Because the signalling cascade is intracellular, we did not expect to observe interactions between extracellular components of the PSA biosynthetic machinery, as supported by the lack of interactions between KpsD and all prey tested (Table S1). In contrast, both homo- and heterotypic interactions were detected between a variety of other *kps* and soluble or membrane-bound *neu* gene products.

### Detection of interacting pairs

The two-hybrid system tests genes or gene fragments fused at the C-terminus of T25 (pKT25 bait derivatives), and prey or quarry constructs fused to the N- or C-terminus of T18 in pUT18 and pUT18C respectively. Plasmids co-transformed into a *cya* background are examined for their Mal phenotypes (white, no interaction; red, strong interaction; pink, weak interaction) on MacConkey-maltose agar plates, and then assayed for beta-galactosidase using the standard reaction with *o*-nitrophenyl β-D-galactoside substrate (Miller, 1972). Backcrossing plasmids into fresh backgrounds confirms that the phenotypes are dependent on the expressed fusions. Positive controls detect interaction between leucine-zipper (Zip) fragments cloned into pKT25 and pUT18C (Karimova *et al.*, 1998). We found that apparent instability of the BTH101 recipient strain sometimes resulted in mixed phenotypes, presumably caused by spontaneous mutation of the reporter gene to cAMP independence or reversion of the *cya* defect. Expressing plasmids in the *recA* strain DHM1 did not solve this inconsistency of the strain background, as expected by the lack of *kps* and *neu* genes in the *E. coli* K-12 genome. Therefore, we constructed *E. coli* K-12 (BW30270) and K1 (EV36) *cya*::*kan* strains EV726 and EV728, respectively, by P1 transduction from strain MER4. After curing the *kan* cassettes with pCP20 (Datsenko and Wanner, 2000), the EV727 and EV729 recipients were used for co-transformation of bait and prey plasmids in the event that wild-type *kps* or *neu* gene products present in the EV36-derived background might influence some interactions. In general, results comparing the two host genetic backgrounds were qualitatively identical, although in some cases, as discussed in the following section, the genetic background had a quantitative effect on the interaction.

Figure S1 shows a schematic diagram for one representative experiment testing interactions between a KpsC bait and a variety of prey plasmids expressed in BTH101, with some prey fusions tested in either of two orientations relative to the *B. pertussis cya* fusion partners. Note that both homo- and heterotypic interactions were detected, suggesting that KpsC has multiple binding domains. Table 2 shows the data for the positively and selected negatively interacting pairs in the EV727 and EV729 backgrounds. Note that NeuS and KpsC interacted strongly, but only when NeuS was the bait (Table 2). This orientation dependency is likely to reflect steric constraints imposed by the *B. pertussis* Cya domains, T25 or T18 (Karimova *et al.*, 1998; 2000). Note that all plasmid fusions were sequenced to verify that translation would be in frame. Although we have not confirmed the positive interactions with antibody pull-down experiments, an immunological study in the *E. coli* K5 system indicated that KpsC-K5 was required for membrane localization of the heparosan (K5 antigen) synthase (Rigg *et al.*, 1998). However, previous results established that NeuS does not require any other *kps* or *neu* gene product for membrane association (Steenbergen *et al.*, 1992), suggesting an alternative function for KpsC in group 2 capsule biosynthesis. Surprisingly, we could not detect an interaction between NeuS and NeuE (Table 2) despite the report that such an interaction might be necessary for polymerization (Andreishcheva and Vann, 2006). However, other interactions between KpsC and itself or KpsE were detected (Table 2), suggesting that KpsC plays a role in connecting PSA export to the polymerase, NeuS, adding support to model 3 (Fig. 2).

**Table 2:** Two-hybrid analysis of selected pair constructs in EV727 and EV729, capsule-negative and capsule-positive, respectively, *E. coli cya* derivatives.

		EV727			EV729		
			
			β-Galactosidase activity^b^			β-Galactosidase activity	
					
Bait/prey plasmids	Gene pairs (bait/prey) tested	Phenotypes on MacConkey^a^	Early	Late	Phenotypes on MacConkey	Early	Late
pKT25/pUT18C	None	−	24 ± 9	23 ± 1	−	31 ± 2	26 ± 2
pKT25-zip/pUT18C-zip	zips	++	830 ± 90	5130 ± 140	++	360 ± 120	4820 ± 450
pSX750/pSX753	*kpsC/kpsC*	+	220 ± 60	540 ± 60	+	200 ± 80	760 ± 180
pSX750/pSX758	*kpsC/kpsE*	+	65 ± 13	120 ± 20	+	230 ± 4	1170 ± 250
pSX750/pSX762	*kpsC/neuS*	−	18 ± 1	ND	−	15 ± 1	ND
pSX751/pSX753	*neuS/kpsC*	++	560 ± 73	4700 ± 70	++	420 ± 80	5540 ± 280
pSX751/pSX758	*neuS/kpsE*	−	58 ± 3	500 ± 75	+	140 ± 19	1050 ± 120
pSX571/pSX767	*neuS/neuE*	−	17 ± 1	ND	−	16 ± 1	ND

a Negative (white); ++, strong positive (deep red); +, weak positive (red). Colonies were scored for Mal phenotypes on MacConkey-maltose agar plates after 2 days of incubation at 30°C.

b β-Galactosidase activity was measured when cells were between A_600_ values of 0.2–0.5 (early) or after overnight incubation (late). Data are given as the mean ± SEM for triplicate experiments.

ND, not determined.

Vann and colleagues previously detected an *in vitro* requirement for KpsC in NeuS-catalysed polymerization (Andreishcheva and Vann, 2006). Furthermore, the estimated size of the radiation-sensitive polymerase complex was 78 kDa larger than the predicted molecular weight of the NeuS monomer (approximately 48 kDa), supporting interaction(s) between NeuS and one or more molecular species totalling less than 48 kDa (Vionnet *et al.*, 2006). Note that NeuS did not interact with itself (Table S1), suggesting that the polymerase might function as a monomer as part of a larger complex. Also note that assaying the polymerase after irradiation only detects the size of the complex necessary for polymerization *in vitro*, indicating that the functional size of the biosynthetic complex (synthesis and export) could be much larger than suggested by enzyme assay. When taken together, the results of our two-hybrid analysis strongly support a direct interaction between KpsC and NeuS. Many other potentially interacting pairs of *kps* or *neu* gene products tested in the two-hybrid system yielded negative results (Table S1), suggesting no or only weak interactions that may have been below the limits of detection. We reasoned that potentially weak interactions might be distinguishable from true negatives if a more sensitive reporter assay was employed.

### Luminescent assay for detection of interacting plasmid pairs

Beta-Glo combines the hydrolysis of a luciferase substrate conjugated to beta-galactoside in a sensitive coupled luminescence assay (Hannah *et al.*, 2003). Relative luminescence units (RLU) are quantified with a luminometer designed for the microtiter-plate format, facilitating data acquisition and replicate analyses. NeuC is an UDP-GlcNAc 2′-epimerase that produces ManNAc as the first committed step in sialic acid biosynthesis. However, the expected homotypic interaction was not statistically significant by standard β-galactosidase assay (Fig. S2A, pair 9), but was observed in the *E. coli* K-12-derived background, EV727 (Fig. S2B, pair 9). Failure to detect a significant homotypic interaction in the EV36-derived background, EV729, could indicate the competitive effect of the wild-type *neuC* gene product on the interaction if it competes with the bait or prey hybrid polypeptide (Fig. S2A). In contrast, pairs 5, 7 and 8 that were negative by standard assay (Table 2) remained so in the luminescence assay (Fig. S2). Positive interactions between pairs 3, 4 and 6 (Fig. S2) confirmed the results of the standard assay (Table 2), although the stronger responses relative to the Zip control (Fig. S2, pair 2) when bait and prey plasmids were coexpressed in the EV36-derived background could indicate enhancement by other *kps* or *neu* gene products, which are absent in EV727 (Fig. S2B). Note that all *neuC*, *neuE* and *neuS* bait or prey plasmids tested in Tables 2, S1 and Fig. S2 complemented their cognate isogenic mutants RS2918, EV725 and EV136, respectively, whereas none of the *kps* constructs complemented theirs. The failure of individual *kps* genes to complement cognate mutants has been previously observed (Vimr *et al.*, 1989), presumably representing the requirement for coexpression of single-copy regions 1 and 3 genes to deliver precise amounts of each region's gene products to the synthetic/export apparatus. DNA sequencing confirmed that the cloning of out-of-frame fusions was not the reason for lack of complementation. Therefore, we carried out deletion analysis of the *kpsC* bait plasmid to define the domains for the homo- and heterotypic interactions of KpsC detected by two-hybrid analysis (Table 2, and Figs S1 and S2).

### Deletion analysis of KpsC bait

**Fig. 9:**
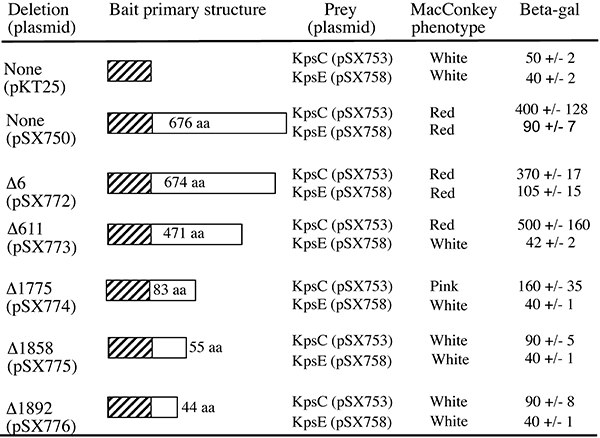
Interacting C-terminal bait deletions of KpsC with full-length KpsC or KpsE prey. The indicated control (pKT25 and pSX750) and deletion bait plasmids were coexpressed with KpsC (pSX753) or KpsE (pSX758) prey plasmids. Beta-galactosidase (Beta-gal) activities were measured in cells grown to mid-log phage in LB medium. The phenotypes on MacConkey-maltose agar plates were scored 2–3 days after transformation. Note that the plate phenotypes and corresponding enzyme activities are only comparable for a given bait–prey combination. Data represent the means ± SEM for three separate experiments. The hatched boxes indicate the N-terminal T25 bait domain while open boxes indicate the relative deletion in base pairs from the C-terminus of KpsC, numbered in amino acid (aa) residues for each deletion as determined by DNA sequencing of the bait constructs. All data were for plasmid pairs expressed in EV727; comparable results were obtained in the EV729 background.

To more precisely define the KpsC binding domains, a series of deletion mutations was prepared by digestion from the 3′ end of *kpsC* in pSX750 with exonuclease III and mung bean exonuclease. The deletions resulted in the removal of 2–633 amino acid residues from the full-length (676 amino acid residues) *kpsC* gene product. As shown in Fig. 9, Δ1775 produced an intermediate phenotype whereas longer deletions in Δ1858 and Δ1892 lost homotypic interactions with the pSX753 *kpsC*-encoded prey. These results strongly implicate an N-terminal region of about 85 amino acid residues as part of the KpsC homotypic binding domain. In contrast, deletion analysis implicated the C-terminal domain as part of the heterotypic KpsE binding domain (Fig. 9). It will be interesting to define the heterotypic binding domain recognizing NeuS, but this will require separate deletion construction because of the orientation dependence on KpsC–NeuS interactions (Table 2). Although the recent purification of the group B meningococcal NeuS orthologue has been shown to polymerize PSA in the absence of other *kps* or *neu* gene products (Freiberger *et al.*, 2007), our current results indicate that capsule biosynthesis requires multiple protein–protein interactions for *in vivo* operation. It is intriguing to speculate that the multiple heterotypic KpsC interactions are required to guide NeuS to the translocation apparatus, with KpsC linking nascent PSA to the KpsE connector. Whether KpsC also has an enzymatic function remains to be determined.

Roberts and colleagues (Rigg *et al.*, 1998) pointed out the apparent *kpsC* gene duplication event that gave rise to homologous N- (Domain A, residues 60–270) and C- (Domain B, residues 387–598) terminal domains with 39.2% similarity over 210 amino acid residues and 58% overall nucleotide identity in the *E. coli* K5 biosynthetic system. Our results indicate that Domain A functions homotypically while Domain B, at least in part, is necessary for heterotypic interaction with KpsE. Interestingly, KpsC-K5 was released by osmotic shock, leading to the speculation that this presumably soluble protein is part of an adhesion complex connecting polysaccharide synthesis and export, potentially involving a KpsE domain (Rigg *et al.*, 1998). Although chemical cross-linking and co-purification with histidine-tagged KpsS-K5 identified apparent interactions between it and KpsM, KpsT, KpsE and KpsD, KpsS-K5 also interacted with other polypeptides whose relevance to capsule biosynthesis is unclear (McNulty *et al.*, 2006), suggesting that chemical cross-linking might produce artefactual results. In contrast, we could not detect interactions between KpsS-K1 and any bait–prey combination tested (Table S1), further suggesting that chemical cross-linking might yield artificial associations or that relevant KpsS interactions are not detectable with the current two-hybrid system. Note that most of our results were obtained with full-length *kps* and *neu* gene fusions. However, the orientation effects on NeuS–KpsC interactions, for example, indicate probable steric constraints interfering with some fusions (Table 2). Therefore, we plan to test shorter fusions of negatively interacting pairs as well as those with demonstrated interactions to more precisely define binding domains and, ultimately, identify specific interacting peptides that should define the complete network of homo- and heterotypic interactions in group 2 capsule systems. This approach should lead to a better understanding of capsule biosynthesis while helping to identify new therapeutic targets in a wide range of encapsulated pathogens expressing group 2 or group 2-like capsules.

## Conclusions

The most important finding of this study is that PSA biosynthesis (synthesis and export) occurs within an intracellular compartment operationally defined as inaccessible to endo-N, a specific depolymerase previously used to investigate PSA in both bacterial and mammalian systems (Vimr *et al.*, 1984). Our approach provides a novel *in vivo* protection assay whereby PSA was detected by K1-specific phage infectivity, electron microscopy and immunodiffusion assessing the processes of translocation, intracellular accumulation of unexported PSA and polymerization respectively. Although we do not know the minimum number of PSA chains necessary for productive lytic phage infection, the number must be no less than 5–10% of the normally present 50 000 chains, as below this minimum number only small plaques are produced because of diminished phage binding (Vimr, 1992; Cieslewicz and Vimr, 1997). Because plaque size was not diminished by endo-N overproduction (Fig. 4), we conclude that more than 5000 PSA chains are synthesized and exported to the outer surface of each cell during capsule biosynthesis in the presence of intracellular endo-N. This conclusion was supported by identical precipitin halos surrounding wild type or cells expressing endo-N (data not shown), which is a reflection of the equivalent amounts of capsular polysaccharide being synthesized and exported during cell growth (Steenbergen and Vimr, 1990). Our previous characterization of the export defects in mutants with regions 1 or 3 defects facilitated detection of endo-N activity *in vivo*. Indeed, our data indicate that there could be two sialisomes: one endo-N-accessible formed by intracellular accumulation of unexported PSA, and the endo-N-inaccessible compartment representing the authentic biosynthetic environment. These results strongly point to model 3 as a dynamic representation of group 2 polysaccharide biosynthesis. (Fig. 2)

We have carried out the first extensive two-hybrid analysis of capsule biosynthesis in any species. The results confirm and extend previous biochemical approaches involving protein cross-linking and immunological detection (Arrecubieta *et al.*, 2001; McNulty *et al.*, 2006), subcellular fractionation and irradiation (Andreishcheva and Vann, 2006; Vionnet *et al.*, 2006), and complementation analyses (Fig. 7). For example, when the biochemical results are combined with our analysis of KpsC interactions, it is clear that KpsC functions in several homo- and heterotypic interactions important to capsule synthesis and export. The N-terminal region necessary for KpsC homotypic interaction has been identified, while a separate C-terminal KpsC domain was shown to mediate heterotypic interaction with KpsE. In contrast, there was no evidence for NeuS oligomerization, although this polypeptide was shown to interact with both KpsC and KpsE, supporting the concept of a dynamic biosynthetic apparatus that couples polymerization to export, in further support of model 3 (Fig. 2). This conclusion implies that the signalling event(s) involved in directed coupling of PSA synthesis and KpsC linking the polymerase to the periplasmic connector, KpsE, could mediate export. Experiments are in progress mapping the NeuS domain that interacts with KpsC. Our results suggest that all groups 2 and 3 polymerases will share similar interactive domains. Finally, despite our inability to detect an interaction between the polymerase and NeuE, suppression of a *neuE* defect by NeuS overproduction supports the conclusion that NeuE is not obligatory for capsule biosynthesis, although it is likely to function in some as yet undefined structural role that is necessary, under the normal single-copy circumstance, for PSA export.

## Experimental procedures

### Bacterial strains, plasmids, phage and growth conditions

The strains, plasmids and phage used in this study are given in Table 3. Bacteria were routinely grown in Lennox-formulated Luria–Bertani (LB) broth purchased from Fisher (Chicago, IL). Kanamycin, ampicillin and tetracycline were used at 50, 100 and 10 μg ml^−1^ of final concentrations respectively. Transductions were carried out using P1*vir* as described previously (Ringenberg *et al.*, 2003). MacConkey agar base with 0.4% maltose was used to screen two-hybrid co-transformants. Plasmid pSX101 was constructed by ligating the BglII-BamHI fragment from plasmid pSX90 into the BamHI site of the pMal-c2 polylinker, which produces an in-frame fusion of the NeuE PIRS domain to MBP. The desired orientation was determined by restriction endonuclease digestion and confirmed by DNA sequencing. Plasmid pSX104 was constructed by ligating the EcoRI-HindIII fragment from plasmid pSX90 into pMal-c2 digested with the same two enzymes. The resulting construct was then linearized with EcoRI, the single-stranded overhangs removed by digestion with mung bean nuclease, and then re-ligated to produce the in-frame fusion, as confirmed by DNA sequencing.

**Table 3:** Bacterial strains, plasmids and phage used in this study.

Strain, plasmid or phage	Genotype or relevant description	Source (or reference)
Bacteria		
BTH101	F^-^*cya-854*, *recA1*, *endA1*, *gyrA96* (*Nal*^*r*^), *thi1*, *hsdR17*, *spoT1*, *rfbD1*, *glnV44*(*AS*)	Karimova *et al.* (1998)
BW30270	*rph*^+^ derivative of MG1655	*E. coli* Genetic Stock Center
DH5α	F^-^, *Φ*80d*lacZ*Δ M15, *recA*1, *endA*1, *gyrA*96, *thi*-1, *hsdR*17 (r^-^_K_, m^+^_K_), *supE*44, *relA*1, *deoR*, Δ(*lacZYA-argF*)U169, *phoA*	Laboratory stock
DHM1	F^-^, *cya-99*, *araD139*, *galE15*, *gal16*, *rpsL1* (*Str*^*r*^), *hsdR2*, *mcrA1*, *mcrB1*	Karimova *et al.* (1998)
EV36	K-12/K1 hybrid	Vimr and Troy (1985)
EV93	EV36 *kpsC::tet*	Vimr *et al.* (1989)
EV94	EV36 *kpsS::tet*	Vimr *et al.* (1989)
EV136	EV36 *neuS::tet*	Steenbergen and Vimr (1990)
EV725	EV36 *neuE::kan*	Vimr and Steenbergen (1993)
EV726	BW30270 *cya::kan*	This study
EV727	BW30270 Δ*cya*	This study
EV728	EV36 *cya::kan*	This study
EV729	EV36 Δ*cya*	This study
HB101	*thi*-1, *hsdS*20 (r_B_^-^,m_B_^-^), *supE*44m *recA*13, *ara*-14, *leuB*6, *proA*2, *lacY*1, *galK*2, *rpsL*20 (str^r^), *xyl*-5, *mtl*-1	Laboratory stock
MER4	F^-^*araD139*, Δ(*argF-lac*)*U169*, *rpsL150*, *relA1*, *fibB5301*, *deoC1*, *ptsF25*, *rbsR*, *cya::kan*	Mark D. Gonzalez
RS2918	EV36 Δ*neuC*	Vann *et al.* 2004
Plasmids		
pUC18	High-copy cloning vector	Laboratory stock
pUC19	pUC18 with inverted multiple cloning site	Laboratory stock
pMAL-c2	Cytoplasmic expression of *malE* fusions	New England Biolabs
pGEM-T Easy	AT cloning vector	Promega
pSR426	6.7 kb EcoRV-BamHI fragment from the KI capsule cluster containing *neuDBACES* in a Bluescript cloning vector	Annunziato *et al.* (1995)
pRep4	*lacI* expression	Qiagen
pEndo-N	Endo-neuraminidase expression	Steenbergen *et al.* (2006)
pSX90	pUC18 with 2.7 kb EcoRI-BamHI fragment from the KI capsule cluster	Steenbergen and Vimr (1990)
pSX91	pUC19 with 2.7 kb EcoRI-BamHI fragment from the KI capsule cluster	Steenbergen and Vimr (1990)
pSX92	pUC18 with 2.3 kb BglII-BamHI fragment from the KI capsule cluster	Steenbergen and Vimr (1990)
pSX93	pUC18 with 3.07 kb HindIII-BamHI fragment from the KI cluster	This study
pSX94	PCR product of *neuE* cloned into pGEM T-Easy	This study
pSX101	pMAL-c2 with the 2.3 kb BglII-BamHI fragment from the K1 cluster, fusing the NeuE PIRS domain to MBP	This study
pSX102	pSX94 with a mutation at aa381 of *neuE*	This study
pSX103	pSX94 with a mutation at aa385 of *neuE*	This study
pSX104	pMAL-c2 with the 2.7 kb EcoRI-BamHI fragment from the K1 cluster, fusing NeuE to MBP	
pKT25	Derivative of pSU40 that carries the T25 fragment of *B. pertussis* adenylate cyclase with a multi-cloning sequence at the 3′ end of T25, also carries a kanamycin-resistance gene	Karimova *et al.* (1998)
pUT18	Derivative of pUC19 that carries the T18 fragment of *B. pertussis* adenylate cyclase with a multi-cloning sequence at the 5′ end of T18, also carries an ampicillin-resistance gene	Karimova *et al.* (1998)
pUT18C	Derivative of pUC19 that carries the T18 fragment of *B. pertussis* adenylate cyclase with a multi-cloning sequence at the 3′ end of T18, also carries an ampicillin-resistance gene	Karimova *et al.* (1998)
pKT25-myc	Derivative of pKT25 with Myc tag	M. Gonzalez
pUT18C-flag	Derivative of pUT18C with Flag tag	M. Gonzalez
pSX750	*kpsC* in pKT25	This study
pSX751	*neuS* in pKT25	This study
pSX752	*kpsD* in pKT25	This study
pSX753	*kpsC* in pUT18	This study
pSX754	*kpsD* in pUT18	This study
pSX755	*neuC* in pUT18	This study
pSX756	*neuC* in pUT18C	This study
pSX757	*kpsD* in pUT18C	This study
pSX758	*kpsE* in pUT18C	This study
pSX759	*neuS* in pKT25-myc	This study
pSX760	*neuE* in pKT25-myc	This study
pSX761	*neuE* in pUT18C	This study
pSX762	*neuS* in pUT18-flag	This study
pSX763	*neuE* in pKT25	This study
pSX764	*kpsS* in pKT25	This study
pSX765	*neuC* in pKT25	This study
pSX766	*neuC* in pKT25-myc	This study
pSX767	*neuE* in pUT18C-flag	This study
pSX768	*kpsM* in pKT25	This study
pSX769	*kpsT* in pKT25	This study
pSX770	*kpsM* in pUT18C	This study
pSX771	*kpsT* in pUT18C	This study
pSX772	Δ*kpsC* in pSX750 missing 9 nucleotides from the C-terminal end	This study
pSX773	Δ*kpsC* in pSX750 missing 614 nucleotides from the C-terminal end	This study
pSX774	Δ*kpsC* in pSX750 missing 1778 nucleotides from the C-terminal end	This study
pSX775	Δ*kpsC* in pSX750 missing 1861 nucleotides from the C-terminal end	This study
pSX776	Δ*kpsC* in pSX750 missing 1895 nucleotides from the C-terminal end	This study
Phage		
P1*vir*	Generalized transduction	Laboratory stock
K1F	K1-specific lytic phage	Vimr *et al.* (1984)

### Cloning *kps* and *neu* genes into two-hybrid plasmids

All bait and prey clones harbour PCR products amplified from boiled EV36. Amplification was carried out using SuperMix High Fidelity (Invitrogen) to eliminate introducing spontaneous mutations into the amplified DNA sequences. In most cases (Table S2), primers were designed to amplify the full-length *kps*- or *neu*-coding sequence with restriction sites in the 5′ end of each primer. Restriction sites were chosen as sites not contained in the gene sequence of interest but present in the multiple cloning site of the two-hybrid plasmid. Sites were designed such that digestion of the PCR product and vector with the specific enzymes, followed by ligation, would yield in-frame fusions of the *kps* or *neu* gene to the T25- or T18-coding sequences in the vectors. Stop codons were included in some primers when cloning into pKT25 or pUT18C, although these vectors also included their own stop codons. PCR products were purified using the ChargeSwitch PCR Clean-Up Kit (Invitrogen) followed by restriction digestion. The digested products were electrophoresed through agarose, the band cut out and the DNA removed from the agarose. This band was then ligated to vector that had been treated with the same restriction enzymes. Insertions were verified by restriction digest. Fusion junctions described in the text were verified, identified by DNA sequencing as previously described (Steenbergen and Vimr, 2003).

### Beta-Glo assay

Bait and prey plasmids were co-transformed into chemically competent EV727 or EV729 by selecting for both ampicillin and kanamycin resistance. Single colonies were inoculated in 2 ml of LB plus the appropriate drugs and grown overnight at 30°C. Three millilitres of fresh media was inoculated with 70 μml of the overnight culture and grown for 2.5–3 h at 30°C. Cultures were then diluted 300-fold and 50 μml portions were added to wells of a white, opaque 96-well microtiter dish (Falcon, number 353296). An equal volume of the Beta-Glo reagent was added to each well and the plate was agitated to mix. The plate was then incubated at room temperature, in the dark, for 30–45 min. Luminescence was quantified on a Wallac Victor2 Multilabel Counter and data expressed as RLU after subtracting background, which was 2000−3000 RLU for medium alone and 3000−6000 RLU for medium plus cells lacking any plasmid. Statistical comparisons were made between cells harbouring vectors without inserts to the Zip control or interacting plasmid pairs after subtraction of the cells alone backgrounds.

### Computer-assisted analysis of NeuE transmembrane regions

Nine different transmembrane (TM)-predictive programs were used to analyse NeuE for potential membrane spanning regions. das (dense alignment surface method) compares dot plots of NeuE against a collection of non-homologous TM proteins using the RreM scoring matrix (Cserzö*et al.*, 1994). hmmtop 2.0 (hidden Markov model for topology prediction) (Tusnády and Simon, 2001) and tmhmm 2.0 (transmembrane helices based on a hidden Markov model) (Krogh *et al.*, 2001) utilize a hidden Markov model distinguishing the inside loop region, inside TM-helix cap, TM helix, outside TM-helix cap and outside loop region, or TM-helix core, caps and loop regions respectively. tmap (transmembrane analysis program) incorporates information from multiple alignments of homologous polypeptides to determine the membrane-spanning segments (Persson and Argos, 1994). pred-tmr2 (prediction of transmembrane regions in proteins) uses the propensities of amino acid residues at the termini of TM helices of proteins compiled by the authors to identify TM segments (Pasquier and Hamodrakas, 1999). TM-Finder uses experimentally derived hydrophobicity and non-polar phase scales to detect TM segments (Deber *et al.*, 2001). TMpred (prediction of transmembrane regions and orientation) uses statistical preference matrices in a data set of membrane proteins compiled by experts to determine likely membrane-spanning regions (Hofmann and Stoffel, 1993). TopPred II (topology prediction of membrane proteins) detects hydrophobic segments using a sliding trapezoid window and evaluates topological models using the positive-inside rule (von Heijne, 1992; Claros and von Heijne, 1994). split 3.5 (University of Split, Croatia) uses integrated scales for amino acids to predict TM regions (Juretíc *et al.*, 1993). Analyses were carried out using the derived primary structure of NeuE (Andreishcheva and Vann, 2006) and the default settings for each programme. The *neuE* sequence chosen for analysis includes the ATG that overlaps 95 bp with the 3′ end of *neuC*.

### Other analytical techniques

The TEM of negatively stained whole mounts or thin sections was carried out as previously described (Cieslewicz and Vimr, 1996; 1997). Maxi-cell analysis and polyacrylamide gel electrophoresis were carried out as described in Cieslewicz *et al.* (1993). For the standard beta-galactosidase assay (Miller, 1976), cells were grown as described above, except that the final dilution was fivefold or 10-fold prior to assay. Data were expressed in standard Miller Units. Western blot analysis was carried out using anti-MBP prepared against purified MBP purchased from New England Biolabs and used according to the manufacturer's instructions.

## Supplementary material

This material is available as part of the online article from: http://www.blackwell-synergy.com/doi/abs/10.1111/j.1365-2958.2008.06231.x (This link will take you to the article abstract).
