# Supplementary material for: Biosynthesis of the Escherichia coli K1 group 2 polysialic acid capsule occurs within a protected cytoplasmic compartment
Source: Mol Microbiol. 2008 Jun;68(5):1252–67. doi: 10.1111/j.1365-2958.2008.06231.x (PMC2408645; doi:10.1111/j.1365-2958.2008.06231.x)
Supplement: Supplementary file 1 [file mmi0068-1252-SD1.pdf]

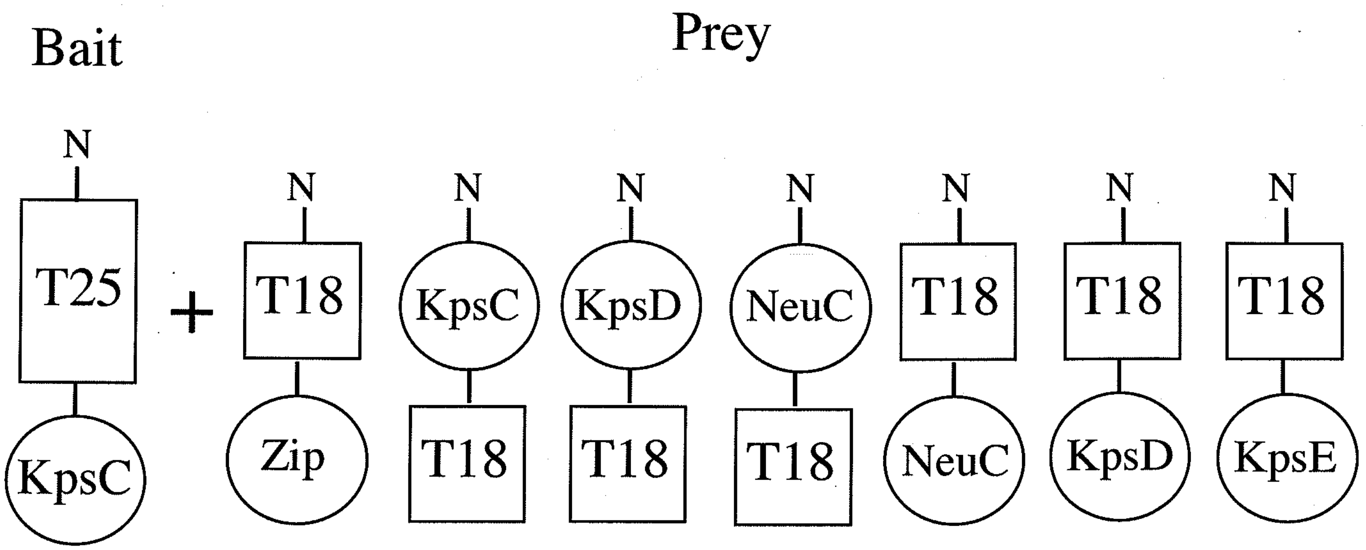

Plasmid	pSX750	pUT18C	pSX753	pSX754	pSX755	pSX756	pSX757	pSX75[illegible]
Beta-gal	-	35	1290	38	36	30	40	137

Fig. S1. Schematic diagram of a representative 2-hybrid experiment. The indicated bait and prey plasmid pairs were cotransformed into strain BTH101. Beta-galactosidase units were determined by the standard assay described in the text.

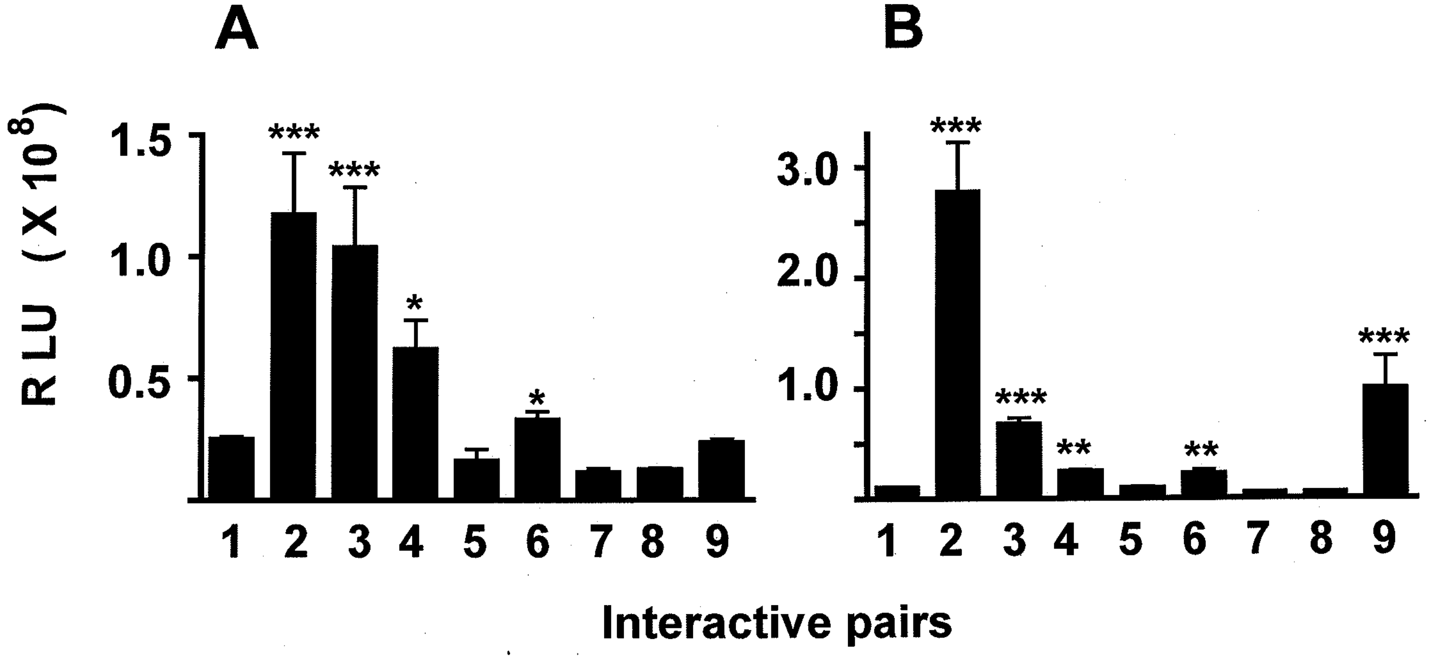

Pair no.	Plasmids	Bait	Prey
1	pKT25 + pUT18C	None	None
2	pKT25-zip + pUT18C-zip	Zip	Zip
3	pSX750 + pSX753	KpsC	KpsC
4	pSX750 + pSX758	KpsC	KpsE
5	pSX750 + pSX762	KpsC	NeuS
6	pSX751 + pSX758	NeuS	KpsE
7	pSX751 + pSX767	NeuS	NeuE
8	pSX760 + pSX762	NeuE	NeuS
9	pSX765 + pSX755	NeuC	NeuC

Fig. S2. Sensitive luminescence assay provides evidence for interactions not detected by the standard beta-galactosidase assay. The indicated plasmid pairs with their bait or prey fusions were analyzed in either EV729 (A) or EV727 (B) host backgrounds. Results show means +/- SEM for four separate experiments each done in duplicate. Data were transformed to their log10 values and analyzed by one-way ANOVA using the Student-Newman-Keuls multiple comparisons test for significant differences between interacting pairs and the vectors-alone control, represented by pair 1. One, two or three asterisks indicate P values <0.05, <0.01 and <0.001, respectively.

Table S1. List of negatively interacting bait and prey combinations.

Plasmid (Bait)	Plasmid (Prey)
pSX750 (*kpsC*)	pSX754 *(kpsD)*
	pSX755 *(neuC)*
	pSX756 *(neuC)*
	pSX757 *(kpsD)*
	pSX761 *(neuE)*
	pSX762 *(neuS)*
	pSX767 *(neuE)*
	pSX777 *(neuO)*
	pSX778 *(kpsF)*
pSX751 *(neuS*)	pSX754 *(kpsD)*
	pSX755 *(neuC)*
	pSX756 *(neuC)*
	pSX757 *(kpsD)*
	pSX758 *(kpsE)*
	pSX761 *(neuE)*
	pSX762 *(neuS)*
	pSX767 *(neuE)*
	pSX777 *(neuO)*
	pSX778 *(kpsF)*
pSX752 *(kpsD)*	pSX753 *(kpsC)*
	pSX754 *(kpsD)*
	pSX755 *(neuC)*
	pSX756 *(neuC)*
	pSX757 *(kpsD)*
	pSX761 *(neuE)*
	pSX762 *(neuS)*
	pSX767 *(neuE)*
	pSX777 *(neuO)*
	pSX778 *(kpsF)*
pSX759 *(neuS)*	pSX753 *(kpsC)*
	pSX754 *(kpsD)*
	pSX755 *(neuC)*
	pSX756 *(neuC)*
	pSX757 *(kpsD)*
	pSX758 *(kpsE)*
	pSX761 *(neuE)*
	pSX762 *(neuS)*
	pSX767 *(neuE)*
pSX760 *(neuE)*	pSX753 *(kpsC)*
	pSX754 *(kpsD)*
	pSX755 *(neuC)*
	pSX756 *(neuC)*

	pSX757 *(kpsD)*
	pSX758 *(kpsE)*
	pSX761 *(neuE)*
	pSX762 *(neuS)*
	pSX767 *(neuE)*
	pSX777 *(neuO)*
	pSX778 *(kpsF)*
pSX763 *(neuE)*	pSX753 *(kpsC)*
	pSX754 *(kpsD)*
	pSX755 *(neuC)*
	pSX756 *(neuC)*
	pSX757 *(kpsD)*
	pSX758 *(kpsE)*
	pSX761 *(neuE)*
	pSX762 *(neuS)*
	pSX767 *(neuE)*
pSX764 *(kpsS)*	pSX753 *(kpsC)*
	pSX754 *(kpsD)*
	pSX755 *(neuC)*
	pSX756 *(neuC)*
	pSX757 *(kpsD)*
	pSX758 *(kpsE)*
	pSX761 *(neuE)*
	pSX762 *(neuS)*
	pSX767 *(neuE)*
	pSX777 *(neuO)*
	pSX778 *(kpsF)*
pSX765 *(neuC)*	pSX753 *(kpsC)*
	pSX754 *(kpsD)*
	pSX756 *(neuC)*
	pSX757 *(kpsD)*
	pSX758 *(kpsE)*
	pSX761 *(neuE)*
	pSX762 *(neuS)*
	pSX767 *(neuE)*
pSX766 *(neuC)*	pSX753 *(kpsC)*
	pSX754 *(kpsD)*
	pSX755 *(neuC)*
	pSX756 *(neuC)*
	pSX757 *(kpsD)*
	pSX758 *(kpsE)*
	pSX761 *(neuE)*
	pSX762 *(neuS)*
	pSX767 *(neuE)*
	pSX777 *(neuO)*

	pSX778 (*kpsF*)
pSX768 (*kpsM*)	pSX753 (*kpsC*)
	pSX754 (*kpsD*)
	pSX755 (*neuC*)
	pSX756 (*neuC*)
	pSX757 (*kpsD*)
	pSX758 (*kpsE*)
	pSX761 (*neuE*)
	pSX762 (*neuS*)
	pSX767 (*neuE*)
	pSX777 (*neuO*)
	pSX778 (*kpsF*)
pSX769 (*kpsT*)	pSX753 (*kpsC*)
	pSX754 (*kpsD*)
	pSX755 (*neuC*)
	pSX756 (*neuC*)
	pSX757 (*kpsD*)
	pSX758 (*kpsE*)
	pSX761 (*neuE*)
	pSX762 (*neuS*)
	pSX767 (*neuE*)

Table S2. Primers used to amplify genes for cloning into two hybrid plasmids.

Baits	Forward primer	Reverse primer
pSX750	5'-TTTTTTG**CTGCAG**GGATGATTGGCATTTAC TCGCCTGGC-3'	5'-TTTTTTTTTT**GGATCC**TTGCCAAATCTGACCTTG CAGAACAT-3'
pSX751, pSX759	5'-AAAAAAAAAG**CTGCAG**GGATGATATTTGAT GCTAGTTTAAAGAAG-3'	5'-AAAAAAAAAT**GGATCC**TTATTACTCCCCCAAGA AAATCCT-3'
pSX752	5'-TTTTTTTTTG**CTGCAG**GGATGAAATTATTTAAA TCAATTTTACTG-3'	5'-TTTTTTTTTT**GGATCC**TTCAAAGACAGAATCACT TTTGCACC-3'
pSX760, pSX763	5'-TTTTTTTTTG**CTGCAG**GGATGCTGTTAGAATT ATTAAGGATTTTA-3'	5'-TTTTTTTTTT**GGATCC**TTAATATCATCTTTCTTAT ACCTTAATAAAAC-3'
pSX764	5'-TTTTTTTTTG**CTGCAG**GGATGCAAGGTAATG CACTAACCGTTTTA-3'	5'-AAAAAAAAAA**GGTACC**CGATATATATTATGTT GGCAGTTTGTTGTGT-3'
pSX765	5'-AAAAAAAAAG**CTGCAG**GGATGAAAAAAATA	5'-AAAAAAAAATT**GGATCC**GAGTCATAACTGGTG

pSX766	TTATACGTAACTGGA-3'	GTACATCCCGG-3'
pSX768	5'-TTTTTTTTTG**CTGCAG**GGATGGCAAGAAGTG GATTTGAAGTT-3'	5'-TTTTTTTTTT**GGATCC**TGATGTCAGCATTGCCTC TTCACG-3'
pSX769	5'-TTTTTTTTTT**CTGCAG**GGATGATTAAGATTGA GAATTTGACG-3'	5'-TTTTTTTTT**GGATCC**TAGGTCTTTTTTGTAATG AGCAATGGCTT-3'

Preys

pSX753	5'-TTTTTTTTT**CTGCAG**GATGATTGGCATTTACTC GCCTGGC-3'	5'-TTTTTTTTTT**GAATTC**GAGCCAAATCTGACCTTGC AGAACAT-3'
pSX754	5'-TTTTTTTTT**CTGCAG**GATGAAATTATTTAAATC AATTTTACTG-3'	5'-TTTTTTTTT**GAATTC**GACAAAGACAGAATCAC TTTTGCACC-3'
pSX755, pSX756	5'-AAAAAAAAAGC**CTGCAG**GATGAAAAAATATT ATACGTAACTGGA	5'-AAAAAAAAAT**GAATTC**GAGTCATAACTGGTGG TACATTCCGGGAT-3'
pSX757	5'-TTTTTTTTT**CTGCAG**GATGAAATTATTTAAATC AATTTTACTG-3'	5'-TTTTTTTTT**GAATTC**GACAAAGACAGAATCACTT TTGCACC-3'
pSX758	5'-AAAAAAAA**CTGCAG**GATGTTGATAAAAGTGAA	5'-AAAAAAAAAT**GAATTC**GAGTCTCGGTGATCTTCA

	GGTCTGCCGTA-3’	ATAACAGC-3’
pSX761	5’-TTTTTTTT**CTGCAG**GATGCTGTTAGAATTATT	5’-TTTTTTTTTT**GGATCC**TTAATATCATCTTTCTTAT
	AAGG-3’	ACCTTAATAAAAC-3’
pSX762	5’-TTTTTTTT**CTGCAG**GATGATATTTGATGCTAGT	5’-TTTTTTTTTT**GGATCC**TTCTCCCCCAAGAAAAT
	TTAAAGAAG-3’	CCTTTTATCG-3’
pSX767	5’-TTTTTTTT**CTGCAG**GATGCTGTTAGAATTA	5’-TTTTTTTTTT**GGATCC**TTAATATCATCTTTCTTATA
	TTAAGG	CCTTAATAAAAC-3’
pSX770	5’-TTTTTTTTT**CTGCAG**GATGGCAAGAAGTGGAT	5’-TTTTTTTTTT**GGATCC**TGATGTCAGCATTGCCTC
	TTGAAGTT-3’	TTCACG-3’
pSX771	5’-TTTTTTTTT**CTGCAG**CATGATTAAGATTGAGA	5’-TTTTTTTTT**GGATCC**TAGGTCTTTTTTGTAATG
	ATTTGACG-3’	AGCAATGGCTT-3’
pSX777	5’-TTTTTTTTT**CTGCAG**GATGTTAAGACTCAAGAC	5’-TTTTTTTTTT**GGATCC**TTGCGTGAGCTTCGCAT
	TCAAGACTC-3’	GATAG-3’
pSX778	5’-TTTTTTTTT**CTGCAG**GATGTCTGAAAGACAT	5’-TTTTTTTTTT**GGATCC**TTTGCCCTTGTTGGTCACC
	TTACCTGATGA-3’	AATA-3’

Nucleotides in bold indicate the restriction sites used for cloning. The start codon for each reading frame is underlined.
